# Long-Term Exposure to Road Traffic Noise and Incident Diabetes: A Cohort Study

**DOI:** 10.1289/ehp.1205503

**Published:** 2012-12-10

**Authors:** Mette Sørensen, Zorana J. Andersen, Rikke B. Nordsborg, Thomas Becker, Anne Tjønneland, Kim Overvad, Ole Raaschou-Nielsen

**Affiliations:** 1 Danish Cancer Society Research Center, Copenhagen, Denmark; 2 Department of Public Health, University of Copenhagen, Copenhagen, Denmark; 3 Department of Environmental Science, Aarhus University, Roskilde, Denmark; 4 Section of Epidemiology, Department of Public Health, Aarhus University, Aarhus, Denmark; 5 Department of Cardiology, Centre for Cardiovascular Research, Aalborg Hospital, Aarhus University Hospital, Aalborg, Denmark

**Keywords:** air pollution, cohort, diabetes, epidemiology, traffic noise

## Abstract

Background: Road traffic noise at normal urban levels can lead to stress and sleep disturbances. Both excess of stress hormones and reduction in sleep quality and duration may lead to higher risk for type 2 diabetes.

Objective: We investigated whether long-term exposure to residential road traffic noise is associated with an increased risk of diabetes.

Methods: In the population-based Danish Diet, Cancer and Health cohort of 57,053 people 50–64 years of age at enrollment in 1993–1997, we identified 3,869 cases of incident diabetes in a national diabetes registry between enrollment and 2006. The mean follow-up time was 9.6 years. Present and historical residential addresses from 1988 through 2006 were identified using a national register, and exposure to road traffic noise was estimated for all addresses. Associations between exposure to road traffic noise and incident diabetes were analyzed in a Cox regression model.

Results: A 10-dB higher level of average road traffic noise at diagnosis and during the 5 years preceding diagnosis was associated with an increased risk of incident diabetes, with incidence rate ratios (IRR) of 1.08 (95% CI: 1.02, 1.14) and 1.11 (95% CI: 1.05, 1.18), respectively, after adjusting for potential confounders including age, body mass index, waist circumference, education, air pollution (nitrogen oxides), and lifestyle characteristics. After applying a stricter definition of diabetes (2,752 cases), we found IRRs of 1.11 (95% CI: 1.03, 1.19) and 1.14 (95% CI: 1.06, 1.22) per 10-dB increase in road traffic noise at diagnosis and during the 5 years preceding diagnosis, respectively.

Conclusion: Exposure to residential road traffic noise was associated with a higher risk of diabetes. This study provides further evidence that urban noise may adversely influence population health.

Exposure to traffic noise has been associated with cardiovascular disease (Babisch 2006; Sørensen et al. 2011a). Noise acts as a stressor and, according to the general stress model, provokes a typical stress response, including hyperactivity of the sympathetic autonomic nervous system and activation of the hypothalamus–pituitary–adrenal axis, resulting in increased blood pressure, heart rate, and high levels of the glucocorticoid cortisol (Ising and Kruppa 2004; Lusk et al. 2004). Also, nighttime exposure to noise at normal urban levels has been associated with sleep disturbances, including short sleep duration and reduced sleep quality, and changes in sleep stages (Miedema and Vos 2007).

Until now research on traffic noise has focused on cardiovascular effects, although given the putative mechanisms of action, traffic noise might also contribute to type 2 diabetes. First, excess of glucocorticoids, as seen in Cushing syndrome, have been found to inhibit insulin secretion by pancreatic β cells and reduce insulin sensitivity in liver, skeletal muscle, and adipose tissue (Mazziotti et al. 2011), as well as increase the risk of diabetes (Chiodini et al. 2005; Clore and Thurby-Hay 2009). Second, experimental reduction in the duration or quality of sleep in human volunteers has been associated with alterations in glucose regulation including a drop in glucose tolerance (Spiegel et al. 1999), increased morning levels of glucose, and decreased levels of insulin (Spiegel et al. 2005) and reduced insulin sensitivity (Stamatakis and Punjabi 2010). Slow-wave sleep, which is associated with inhibition of cortisol secretion, decreased sympathetic nervous system activity, increased vagal tone, and stimulation of growth hormone release, is especially important for glucose regulation (Spiegel et al. 2009). A 90% reduction in slow-wave sleep caused by acoustic stimuli has been associated with decreased glucose tolerance and reduced insulin sensitivity (Buxton et al. 2010; Tasali et al. 2008). Third, hormones responsible for appetite regulation have been found to be affected by sleep reduction, with decreased leptin levels and elevated ghrelin levels resulting in up-regulation of appetite, which in turn may result in higher body mass index (BMI) and an increased risk of diabetes (Spiegel et al. 2004; Taheri et al. 2004).

Epidemiological studies also support a relationship between sleep disturbances and diabetes. In 2010, Cappuccio et al. conducted a meta-analyses of 10 prospective epidemiological studies that investigated the relationship between quantity and quality of sleep and the incidence of type 2 diabetes (Cappuccio et al. 2010). Their analysis of a total combined study sample of > 100,000 participants and 3,586 incident cases indicated that both the quality and quantity of sleep consistently and significantly predicted the risk of type 2 diabetes.

The aim of the present study was to investigate the hypothesis that exposure to residential road traffic noise increases the risk of incident diabetes.

## Methods

*Study population.* The study was based on the Danish Diet, Cancer and Health cohort (Tjønneland et al. 2007). In total, 57,053 of 160,725 residents of Copenhagen or Aarhus who were 50–64 years of age without a history of cancer were enrolled into the cohort between 1993 and 1997. Participants had to have been born in Denmark. At enrollment, each participant completed self-administered, interviewer-checked questionnaires covering food intake, lifestyle habits including detailed information on present and previous smoking and physical activity, health status, and social factors. Height, weight, and waist circumference were measured by trained staff members according to standardized protocols. The study was conducted in accordance with the Helsinki Declaration and approved by the local ethical committees (Copenhagen and Frederiksberg), and all participants provided written informed consent.

*Identification of outcome.* Incident diabetes cases diagnosed between baseline and death, emigration, or the end of follow-up (27 June 2006) were identified by linking the unique personal identification number of each cohort member to the Danish National Diabetes Registry (NDR) (Carstensen et al. 2008). The NDR was established in 2006 by the National Board of Health by linking the following nationwide registries and data: the National Hospital Registry, for hospital discharge diagnoses since 1977; the National Health Insurance Registry, for information on all services provided by general and specialist practitioners since 1973; and the Register of Medicinal Product Statistics, for information on all prescriptions dispensed at Danish pharmacies since 1993. Inclusion criteria for the NDR were as follows: a hospital discharge diagnosis of diabetes in the National Patient Register {*International Classification of Diseases, 10th Revision* [ICD-10; World Health Organization (WHO) 1993]: DE10–14, DH36.0 and DO24}; National Health Insurance Registry information indicating podiatry (chiropody) for diabetic patients, five blood glucose measurements within 1 year, or two blood glucose measurements per year for 5 consecutive years; or > 1 purchase of insulin or oral glucose-lowering drugs within 6 months registered in the Register of Medicinal Product Statistics. The registry has been reported to have a positive predictive value of 89% (Carstensen et al. 2011). Among the 3,869 cases in the present study 55% met more than one inclusion criterion. The date of inclusion into the NDR has been found to be well defined only for persons entering after 1 January 1995 (Carstensen et al. 2008), so the incidence of diabetes is defined as the date of the earliest record in the NDR after 1 January 1995 and before 27 June 2006. Participants were excluded from the present analysis if they were diagnosed with diabetes before 1995, or were diagnosed before baseline if enrolled after 1995. In addition, we excluded participants diagnosed with cancer before baseline. We also applied a stricter incidence definition by excluding individuals registered in the NDR solely because of a history of blood glucose tests.

*Exposure assessment.* A complete residential address history between 1988 and diagnosis (i.e., the date of the first record in the NDR, as defined above) or censoring was collected for 93% of the cohort members. Road traffic noise exposure was calculated for the years 1990, 1995, 2000, and 2005 using SoundPLAN (version 6.5; http://www.soundplan.dk/) for all residential addresses at which cohort members had lived between 1988 and diagnosis/censoring. This noise calculation program implements the joint Nordic prediction method for road traffic noise, which has been the standard method for noise calculation in Scandinavia since the first version of the method was published in 1981 (Bendtsen 1999).

The input variables for the noise model were as follows: point for noise estimation [geographical coordinates and height calculated as 2 m + 3 m × (floor level–1)]; road links with information on annual average daily traffic, vehicle distribution (of light and heavy vehicles), travel speed, and road type (motorway, express road, road wider than 6 m, road narrower than 6 m and wider than 3 m, and other road); and building of polygons for all buildings, including information on building height. We obtained traffic counts for all Danish roads with > 1,000 vehicles per day from a national road and traffic database (Jensen et al. 2009). This database is based on a number of different traffic data sources ranked as follows:

traffic data from the 140 Danish municipalities with most residents, covering 97.5% of the addresses included in the present study; included roads typically have > 1,000 vehicles per day and are based on traffic counts as well as estimated/modeled numbers, and traffic data represent the period from 1995–1998.
traffic data from a central database covering all the major state and county roads.
traffic data for 1995–2000 for all major roads in the Greater Copenhagen Area.
smoothed traffic data for 1995 for all roads based on a simple method where estimated figures for distribution of traffic by road type and by urban/rural zone are applied to the road network and subsequently calibrated against known traffic data at county level (traffic performance).

We assumed that the terrain was flat, which is a reasonable assumption in Denmark, and that urban areas, roads, and areas with water were hard surfaces, whereas all other areas were acoustically porous. No information was available on noise barriers or road surfaces. Road traffic noise was calculated as the equivalent continuous A-weighted sound pressure level (L_Aeq_) at the most exposed facade of the dwelling at each address for the day (L_d_; 0700–1900 hours), evening (L_e_; 1900–2200 hours), and night (L_n_; 2200–0700 hours), and was expressed as L_den_ (day, evening, night) by applying a 5-dB penalty for the evening and a 10-dB penalty for the night. Similar to a previous study, all values < 42 dB were set to 42 dB (Selander et al. 2009), because we considered this a lower limit of ambient noise.

Railway noise exposure was calculated as the A-weighted level (L_Aeq_, 24 hr) outside the most exposed facade using the joint Nordic prediction method for railway noise (Lathi 1984), based on general information about rail traffic in 1993–2000. The model calculated exposures in the range of 60–80 dB. The estimated level of railway noise is assumed to be representative for the study period (1990–2006), because neither high-speed rail tracks nor other new rail tracks were in operation in Denmark, and cargo rail traffic was stable. Screening by designated noise screens or buildings was not considered. The noise impact from all Danish airports and airfields was determined from information about noise zones (5-dB categories) obtained from local authorities. The programs DANSIM (Danish Airport Noise Simulation Model) and INM3 (Integrated Noise Model), which fulfill the joint Nordic criteria for air traffic noise calculations, were used (Liasjø and Granøien 1993). The curves for railway and aircraft noise were transformed into digital maps and linked to each address by geocodes.

The concentration of nitrogen oxides (NO_x_) in the air was calculated using the Danish AirGIS modeling system (http://www.dmu.dk/en/air/models/airgis/) for each year (1988–2006) at each address at which the cohort members had lived. AirGIS allows calculation of air pollution at a location as the sum of local air pollution from traffic in the streets based on the Operational Street Pollution Model; the urban background contribution based on an area source dispersion model; and a regional background contribution (Berkowicz et al. 2008). Input data for the AirGIS system included traffic data for individual road links (same input data as described for the noise modeling), emission factors for the Danish car fleet, street and building geometry, building height, and meteorological data (Jensen et al. 2001). The AirGIS system has been successfully validated and applied in several studies (Ketzel et al. 2011; Raaschou-Nielsen et al. 2011). For example, AirGIS-modeled estimates were highly correlated with 1-month mean concentrations of NO_x_ measured over an 8-year period (1998–2005) in a busy street in Copenhagen (Jagtvej; 25,000 vehicles/day, street canyon), with a correlation coefficient of 0.88 (Ketzel et al. 2011).

*Statistical methods.* The analyses were based on a Cox proportional hazards model with age as the underlying time metric (Thiebaut and Benichou 2004). This ensured comparison of individuals of the same age. We used left truncation at age of enrollment, so that people were considered at risk from the date of enrollment into the cohort, and right censoring at the age of diabetes diagnosis (event), death, emigration, or end of follow-up (27 June 2006), whichever came first. Exposure to road traffic noise and NO_x_ were modeled as time-weighted averages for the preceding 5 years (taking all present and historical addresses during that period into account), or as the average yearly exposure at the current residence. These exposures (1 and 5 years) were entered as time-dependent variables into the statistical risk model, so exposure was estimated for all cohort members who were at risk of diagnosis at exactly the same age as each case at diagnosis.

Incidence rate ratios (IRRs) for diabetes in association with road traffic noise were calculated for *a*) average yearly road traffic noise at the current residence, and *b*) time-weighted mean road traffic noise during the previous 5 years. Estimates were adjusted for potential confounders defined *a priori*, most of which were classified at baseline, including sex, smoking status (never, former, current), smoking intensity (grams tobacco/day), smoking duration (years), environmental tobacco smoke (yes/no), intake of fruit (grams/day), intake of vegetables (grams/day), intake of saturated fat (grams/day), length of school attendance (< 8, 8–10, > 10 years), socioeconomic status of the participant’s municipality (or district for Copenhagen; 10 districts in total) classified as low, medium low, medium high, or high based on municipality/district-level information on education, work market affiliation and income, occupational status (employed, unemployed/retired), alcohol consumption (yes/no), alcohol intake (grams/day), body mass index (BMI; kilograms per meter squared), waist circumference (centimeters), sport during leisure time (0, 0.5–1.5, > 1.5 hr/week), walking during leisure time (≤ 1, 1.5–5, > 5 hr/week), and bicycling during leisure time and transport to work (0, 0.5–2, > 2 hr/week). Also, we adjusted for calendar year to account for time trends in exposures and the outcome. The remaining covariates were specific for each address, including railway and airport noise [> 60 dB (yes/no)], and air pollution (NO_x_, micrograms per cubic meter, calculated as the yearly mean at the current residential address or the time-weighted mean for the previous 5 years, consistent with the time period modeled for road traffic noise). Potential modification of the association between road traffic noise and diabetes by baseline characteristics and age at diagnosis were evaluated by introducing interaction terms into the model, and were tested by the Wald test.

In addition to modeling road traffic noise as a continuous variable, we estimated associations with six noise exposure categories [52–55 dB (714 cases), 55–58 dB (657 cases), 58–61 dB (518 cases), 61–64 dB (474 cases), 64–67 dB (330 cases), > 67–70 dB (498 cases)] relative to a common reference category (≤ 52 dB, 678 cases). An increase of 3 dB corresponds to a doubling in acoustical energy. The cut point of 52 dB for the reference group was chosen to obtain a stable reference group and at the same time be able to evaluate the dose–response relationship in a large part of the exposure span.

We also estimated IRRs for diabetes in association with exposure to railway noise of ≥ 60 dB.

The assumption of linearity of L_den_ in relation to risk of diabetes was evaluated both visually and by formal testing with linear spline models with boundaries placed at the nine deciles for cases. L_den_ did not deviate from linearity (*p* = 0.18). The procedure PHREG in SAS version 9.1 (SAS Institute Inc., Cary, NC, USA) was used for the statistical analyses. The graphical presentation of a functional form of an association between L_den_/NO_x_ and diabetes was produced using restricted cubic spline in the design library (R 2.13.1 statistical software; http://cran.r-project.org/bin/windows/base/old/2.13.1/).

## Results

Among the 57,053 cohort participants, 571 were excluded due to a diagnosis of cancer before enrollment. A complete residential address history from January 1988 to the event or censoring date was collected for 53,673 of the 56,482 remaining participants. Of these we excluded 1,136 participants with self-reported diabetes at baseline, 170 with a diabetes record in NDR before baseline, 10 with a diabetes diagnosis in the NDR between baseline and 1 January 1995, and 2,170 participants with missing data for one or more covariates. Diabetes was diagnosed in 3,869 of the 50,187 eligible participants during an average follow up of 9.6 years, including 1,117 registered in the NDR based only on blood glucose measurement. Excluding these cases for a more strict definition of diabetes resulted in 2,752 cases.

Compared with the cohort as a whole, cases were more likely to be men, were older at enrollment, had higher BMI and waist circumference, had lower education, smoked more, were more exposed to environmental tobacco smoke, ate less fruit and vegetables, were less physically active, and were exposed to higher levels of road traffic noise (Table 1). There was a positive correlation between L_den_ and NO_x_ during the study period (*R*_S_ = 0.62).

**Table 1:** Baseline characteristics of the Diet, Cancer and Health cohort by incident diabetes status at follow-up.

Characteristic at enrollment	Total cohort (n = 50,187)	All diabetes cases (n = 3,869)
Men (%)	47.1	56.1
Age (years)	56.1 (50.7–64.2)	57.3 (50.9–64.4)
BMI (kg/m2)	25.5 (20.4–33.2)	28.6 (22.3–37.9)
Waist circumference (cm)	88 (69–110)	98 (75–120)
Years of school attendance (%)
≤ 7	32.8	39.6
8–10	46.5	44.8
> 10	20.8	15.6
Occupational status (%)
Employed	78.3	72.3
Unemployed/retired	21.8	27.7
Socioeconomic status (%)a
Low	15.9	17.4
Medium low	46.4	44.2
Medium high	16.3	16.1
High	21.4	22.4
Smoking status (%)
Never	36.2	30.2
Former	27.5	30.3
Current	36.3	39.6
Smoking duration (years)b	33.0 (7.0–46.0)	34.0 (9.0–47.0)
Smoking intensity (g/day)b	14.7 (3.75–34.1)	17.0 (4.94–36.3)
Environmental tobacco smoke (%)	63.9	68.3
Drink alcohol (%)	97.8	97.0
Alcohol intake (g/day)	13.4 (1.15–64.5)	13.3 (0.79–72.4)
Fruit intake (g/day)	169 (27.0–523)	162 (24.0–509)
Vegetable intake (g/day)	161 (49.0–363)	144 (41.9–348)
Saturated fat intake (g/day)	31.1 (15.8–55.1)	31.3 (15.7–56.8)
Sport during leisure time (%)
No	45.5	55.9
Yes, ≤ 1.5 hr/week	25.5	20.6
Yes, > 1.5 hr/week	29.0	23.4
Bicycling during leisure time (%)
No	31.8	38.3
Yes, ≤ 2 hr/week	36.0	33.7
Yes, > 2 hr/week	32.3	28.0
Walking during leisure time (%)
0–1 hr/week	22.1	23.1
1.5–5 hr/week	54.2	51.8
> 5 hr/week	23.7	25.1
Road traffic noise (dB)	56.4 (48.5–70.0)	57.1 (48.5–70.7)
Air pollution, NOx (µg/m3)	20.8 (14.4–87.3)	20.8 (14.4–95.4)
Values are medians (5th–95th percentiles) unless otherwise stated. aSocioeconomic status of municipalities based on municipality information on education, work market affiliation, and income. bAmong present and former smokers.		

**Figure 1:**
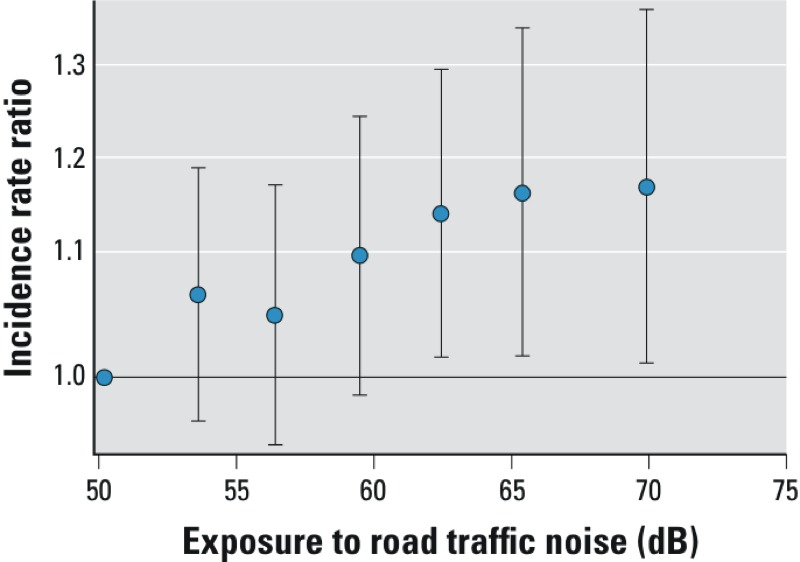
Association between exposure to road traffic noise (L_den_) at the residence at the time of diagnosis and all incident diabetes adjusted for age; sex; BMI; waist circumference; smoking status, duration, and intensity; environmental tobacco smoke; intake of fruits, vegetables, saturated fat, and alcohol; sport; bicycling and walking; school attendance; occupational status; municipality socioeconomic status; railway and airport noise; air pollution; and calendar year. The vertical whiskers show incidence rate ratios (IRR) with 95% CIs at the median of six exposure categories (52–55, 55–58, 58–61, 61–64, 64–67, > 67 dB) when compared with the reference category of ≤ 52 dB.

A 10-dB higher level of exposure to road traffic noise at the current residence and during the previous 5 years was associated with statistically significant 8% (95% CI: 1.02, 1.14) and 11% (95% CI: 1.05, 1.18) higher risk of incident diabetes, respectively, based on fully adjusted models (model 3, Table 2). The analysis of road traffic noise as a categorical variable was generally consistent with a linear exposure–response relationship (Figure 1). When the stricter definition of diabetes was used, the IRRs were slightly higher (Table 2). Adjusting for NO_x_ resulted in small increases in the effect estimates (model 2 vs. model 3), but exposure–response curves based on restricted cubic splines were comparable before and after adjustment for NO_x_ [see Supplemental Material, Figure S1 (http://dx.doi.org/10.1289/ehp.1205503)].

**Table 2:** IRRs (95% CIs) of diabetes per 10-dB higher level of exposure to road traffic noise based on 50,187 cohort participants.

Exposure to road traffic noise Lden (per 10 dB)	Cases (n)	Model 1: Adjusted for age	Model 2: Adjusted for age, sex, lifestyle confounders,a socioeconomic confounders,b calendar year, railway and airport noise	Model 3: model 2 + residential exposure to NOxc
All diabetesd
Lden at diagnosis	3,869	1.09 (1.04, 1.14)	1.06 (1.01, 1.11)	1.08 (1.02, 1.14)
Lden 5 years preceding diagnosis	3,869	1.11 (1.06, 1.16)	1.08 (1.02, 1.13)	1.11 (1.05, 1.18)
Confirmed diabetesd
Lden at diagnosis	2,752	1.14 (1.08, 1.20)	1.08 (1.02, 1.14)	1.11 (1.03, 1.19)
Lden 5 years preceding diagnosis	2,752	1.16 (1.10, 1.23)	1.09 (1.03, 1.16)	1.14 (1.06, 1.22)
aBMI; waist circumference; smoking status; smoking duration; smoking intensity; environmental tobacco smoke; intake of saturated fat, fruits, vegetables, and alcohol; sport; bicycling and walking during leisure time. bLength of school attendance, occupational status and municipality SES. cExposure calculations for NOx follow the exposure calculation for road traffic noise, such that models of Lden at diagnosis are adjusted for NOx at diagnosis and models of Lden during the 5 years preceding diagnosis are adjusted for NOx during the 5 years preceding diagnosis. dAll diabetes: all original criteria in the National Diabetes Registry: hospital admission, medication, reimbursement for chiropody due to diabetes, or glucose blood tests; confirmed diabetes: exclusion of cases only included in the National Diabetes Registry based on blood glucose tests.				

We found no significant effect modification (all Wald *p*-values > 0.05), although there were indications of a stronger association with road traffic noise among women (1.11; 95% CI: 1.03, 1.20) compared with men (1.05; 95% CI: 0.98, 1.13) and among participants > 65 years of age (1.12; 95% CI: 1.03, 1.21) compared with younger participants (1.05; 95% CI: 0.98, 1.13). In addition, road traffic noise was not associated with diabetes in participants with > 10 years of education (1.00; 95% CI: 0.88, 1.14), but was associated with diabetes among those with less education (Table 3).

**Table 3:** Modification of associations between yearly road traffic noise (per 10 dB) at the residential address at diagnosis and incident diabetes (all) by baseline characteristics and age at diagnosis.

Covariates	Cases (n)	IRR (95% CI)a	p-Interaction
Sex			0.24
Men	2,172	1.05 (0.98, 1.13)
Women	1,697	1.11 (1.03, 1.20)
Age at diabetes diagnosis (years)			0.21
< 65	2,323	1.05 (0.98, 1.13)
≥ 65	1,546	1.12 (1.03, 1.21)
BMI (kg/m2)			0.95
Normal and underweight (< 25 kg/m2)	727	1.09 (0.97, 1.23)
Overweight (25–30 kg/m2)	1,704	1.07 (0.99, 1.16)
Obese (≥ 30 kg/m2)	1,438	1.07 (0.99, 1.17)
Years of education			0.39
≤ 7	1,553	1.10 (1.02, 1.20)
8–10	1,734	1.08 (1.00, 1.17)
> 10	602	1.00 (0.88, 1.14)
Smoking status			0.35
Never	1,167	1.11 (1.01, 1.22)
Former	1,172	1.02 (0.93, 1.12)
Present	1,530	1.10 (1.01, 1.19)
Cardiovascular diseaseb			0.42
Yes	199	0.99 (0.80, 1.22)
No	3,664	1.08 (1.02, 1.15)
Residential exposure to NOx			0.66
< 14.1 µg/m3	984	1.11 (0.97, 1.26)
14.1–17.7 µg/m3	1,004	1.09 (0.97, 1.22)
17.7–25.1 µg/m3	913	1.12 (0.99, 1.27)
≥ 25.1 µg/m3	968	1.02 (0.91, 1.14)
aAdjusted for age; sex; BMI; waist circumference; smoking status; smoking duration; smoking intensity; environmental tobacco smoke; intake of fruits, vegetables, saturated fat, and alcohol; sport; bicycling and walking during leisure time; length of school attendance; occupational status; municipality socioeconomic status; railway and airport noise; exposure to air pollution (NOx); and calendar year. bStroke and/or myocardial infarction at enrollment.			

We found no associations between exposure to railway noise of ≥ 60 dB and risk of all diabetes (IRR = 0.97; 95% CI: 0.89, 1.05) or confirmed diabetes (IRR = 1.01; 95% CI: 0.91, 1.11).

## Discussion

In this study, residential exposure to road traffic noise was associated with a higher risk of incident diabetes, with stronger associations at higher levels of exposure. Associations also were slightly stronger with longer-term exposure (5 years) than with shorter-term exposure (1 year).

The strengths of our study include the prospective design, the large number of cases, access to residential address histories, and diagnosis of incident diabetes using a nationwide register. Because all Danish citizens have free access to the health care system, capture of diabetes cases in the registry is assumed to be relatively unrelated to socioeconomic status. Another strength is adjustment for air pollution, which correlates with road traffic noise and has been associated with diabetes (Andersen et al. 2012; Coogan et al. 2012). In the present study, road traffic noise and NO_x_ were moderately correlated, with 38% of the variation in noise predicted by air pollution exposure. We found that exposure to road traffic noise was significantly associated with incident diabetes both before and after adjustment for air pollution, suggesting an independent effect of road traffic noise.

Some limitations also need to be considered. The diabetes registry does not contain information on whether the diabetes is of type 1 or type 2. Type 2 diabetes normally constitutes 90–95% of all diabetes cases. However, at enrollment all participants in the cohort reported whether they had been diagnosed with diabetes before enrollment from 50 years of age, and because type 1 diabetes is most commonly developed during childhood, most cases with type 1 diabetes would have been excluded from our study. Approximately 60% of individuals with diabetes among a Danish population 30–60 years of age were reported to be unaware of their disease (Glumer et al. 2003). Therefore, the date of diagnosis captured in the diabetes registry will, for many cases, only poorly reflect when they actually developed diabetes.

Noise exposure was estimated based on modeled values. The level of traffic noise varies over very short time periods due to, for example, movement of vehicles relative to the observer, and weather conditions also may strongly influence the propagation of traffic noise. It is therefore extremely difficult, if not impossible, to estimate reliable long-term noise exposure based on direct measurements. During the last four decades, increasingly accurate and reliable prediction methods for traffic noise have been developed. Nonetheless, although the Nordic prediction method has been used for many years, estimation of noise is inevitably associated with some degree of uncertainty. Inaccurate input data may contribute to exposure misclassification, but because the noise model does not distinguish between cases and noncases, such misclassification is likely to be nondifferential, and this, in most situations, would bias the relative risk estimate toward the null value. Also, we had no information on bedroom location, noise from neighbors and ventilation, or hearing impairment, all of which might influence exposure to noise. A previous study investigating effects of road traffic noise on myocardial infarction found a stronger association when several of these factors were considered (Selander et al. 2009), suggesting that the effect of noise might be underestimated in the present study.

It is estimated that > 30% of the population of the European Union is exposed to road traffic noise levels at their residence that exceed the WHO guidance limit for noise (Berglund et al. 1999; WHO 2009). Research on the health effects of exposure to traffic noise has focused on cardiovascular diseases, including three studies based on the cohort evaluated in the present study (Sørensen et al. 2011a, 2011b, 2012). The results have supported an effect of traffic noise on blood pressure (Dratva et al. 2011; Sørensen et al. 2011b), myocardial infarction (Babisch 2008; Sørensen et al. 2012), and stroke (Sørensen et al. 2011a). The present study suggests that exposure to road traffic noise may also be involved in the development of diabetes, which, given the assumed mechanisms of action of traffic noise, is supported by studies of effects of excess cortisol and sleep disturbances on glucose tolerance, insulin sensitivity and hormones responsible for regulation of appetite, and the risk of diabetes (Buxton et al. 2010; Chiodini et al. 2005; Clore and Thurby-Hay 2009; Mazziotti et al. 2011; Spiegel et al. 1999, 2005, 2009; Tasali et al. 2008, 2009). These proposed mechanisms might be more important for the development of type 2 diabetes than for cardiovascular disease. Because type 2 diabetes develops over many years (Expert Committee on the Diagnosis and Classification of Diabetes Mellitus 2003) and is often diagnosed years after actual onset (Glumer et al. 2003), we would expect long-term exposure to road traffic noise to be more strongly associated with diabetes than shorter-term exposure, as our results suggest. However, only 28% of participants moved during follow-up in our study (1993–2006) causing a strong correlation between recent and more distant exposure, and it is therefore difficult to separate the effect of recent and distant noise exposure in relation to diabetes.

Our results also suggest that there was no association between road traffic noise and diabetes among participants with > 10 years of education, in contrast with participants who had less education. A possible explanation is that more educated participants may live in larger houses or flats than less educated participants, and therefore may be more likely to have the option to chose a bedroom oriented away from a busy street, resulting in lower exposure to road traffic noise during sleep and differential misclassification of exposure according to education. Another possible explanation is that the observed association between road traffic noise and diabetes among less-educated participants could reflect residual confounding by socioeconomic factors that were not accounted for in our analyses. The assumption that the highest educated were exposed to lower levels of traffic noise than the less-educated participants, combined with residual confounding by socioeconomic factors such as physical activity, could result in false positive associations between noise and diabetes among the lowest educated. On the other hand, differences in exposure to traffic noise according to socioeconomic status might not be pronounced because many highly educated people in Denmark live in central urban areas, evidenced by very high property prices in the inner cities of Copenhagen and Aarhus, with relatively high traffic noise. In the present study we estimated only small differences in road traffic noise exposure according to education (averages of 58.4, 58.1, and 57.5 dB for low, medium, and high education, respectively), which suggests that residual socioeconomic confounding is not a major problem in the present study. Residual confounding by dietary factors not accounted for might also be an issue, though adjusting for potential confounders related to socioeconomic status and diet, such as years of school attendance, BMI, physical activity, occupational status, and intake of fruit, vegetables, and saturated fat had little effect on estimated associations.

We found no association between exposure to railway noise and diabetes, consistent with previous studies reporting that road traffic noise is associated with more sleep disturbance than railway noise (Miedema and Vos 2007). However, exposure estimates for railway noise, which was classified in 5-dB categories for levels ≥ 60 dB only, were less accurate than estimates of road traffic noise exposure. Furthermore, in contrast to the road traffic noise model, the railway noise estimation included no information on screening by buildings.

## Conclusions

This study provides further evidence that urban noise may adversely influence population health. We found a statistically significant positive association between long-term exposure to road traffic noise at the residence and the risk of incident diabetes. The results suggest that reducing population exposure to road traffic noise may improve health.

## Supplemental Material

(111 KB) PDF
