# Supplementary material for: Long-Term Exposure to Road Traffic Noise and Incident Diabetes: A Cohort Study
Source: Environ Health Perspect. 2012 Dec 10;121(2):217–22. doi: 10.1289/ehp.1205503 (PMC3569689; doi:10.1289/ehp.1205503)
Supplement: (111 KB) PDF [file ehp.1205503.s001.pdf]

Supplemental Material

Long-Term Exposure to Road Traffic Noise and Incident Diabetes: A Cohort Study

Mette Sørensen, Zorana J Andersen, Rikke B Nordsborg, Thomas Becker, Anne Tjønneland,

Kim Overvad, Ole Raaschou-Nielsen

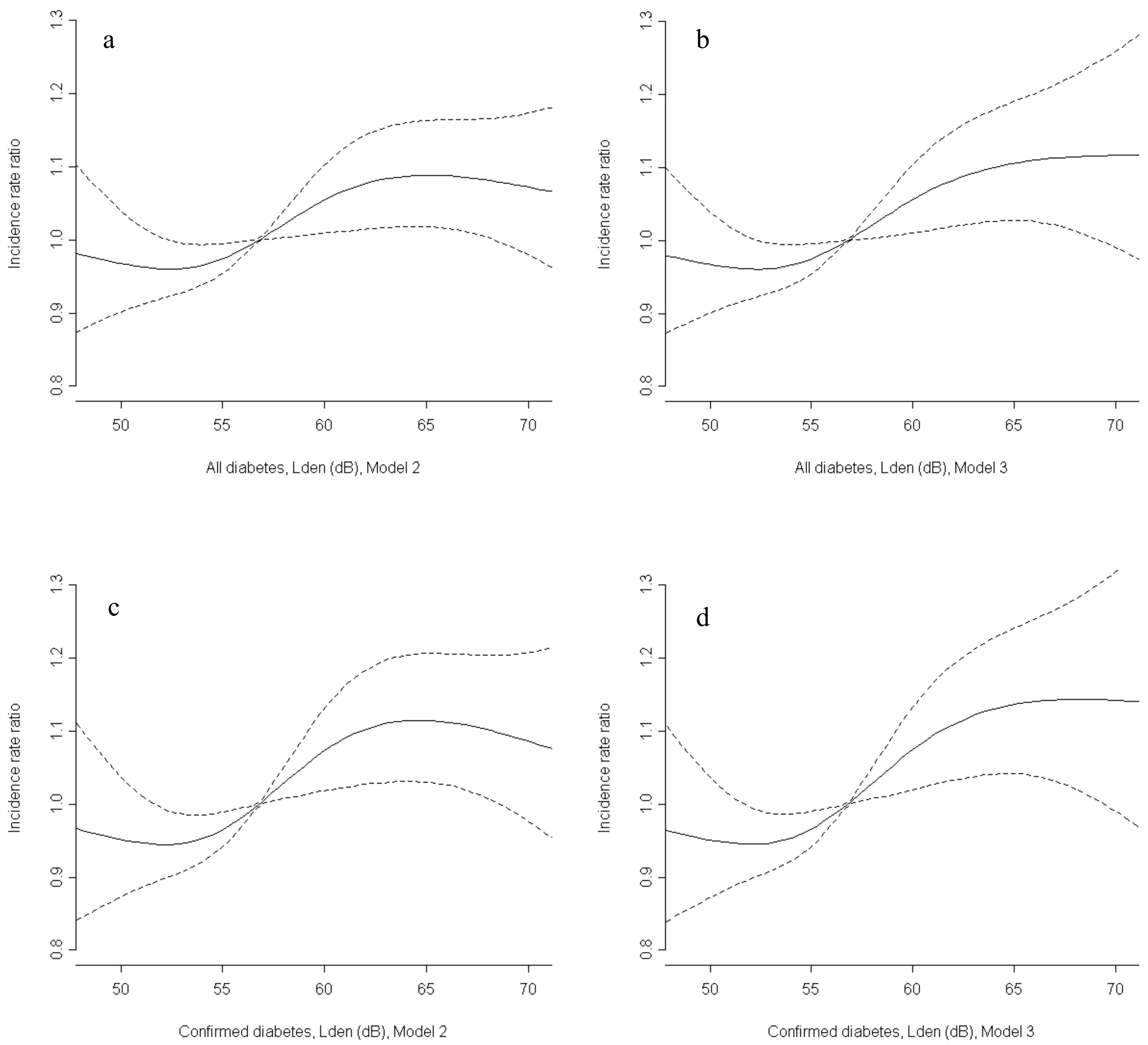

Figure S1

Incidence rate ratios (solid lines) with 95% confidence intervals (dashed lines) for the relation between exposure to L_{den} (year preceding diagnosis) and risk for a) and b) all incident diabetes without and with adjustment for exposure to NO_x, respectively, and c) and d) confirmed incident diabetes without and with adjustment for exposure to NO_x, respectively, all adjusted for age, sex, BMI, waist circumference, smoking status, smoking duration, smoking intensity, environmental tobacco smoke, intake of fruits, vegetables, saturated fat and alcohol, sport, bicycling and walking during leisure time, length of school attendance, occupational status, municipality SES, railway and airport noise and calendar-year.
